# Omics-Based Approach Reveals Complement-Mediated Inflammation in Chronic Lymphocytic Inflammation With Pontine Perivascular Enhancement Responsive to Steroids (CLIPPERS)

**DOI:** 10.3389/fimmu.2018.00741

**Published:** 2018-04-23

**Authors:** Morten Blaabjerg, Anne Louise Hemdrup, Lylia Drici, Klemens Ruprecht, Peter Garred, Romana Höftberger, Bjarne W. Kristensen, Daniel Kondziella, Tobias Sejbaek, Soren W. Hansen, Helle H. Nielsen, Pia Jensen, Morten Meyer, Friedemann Paul, Hans Lassmann, Martin R. Larsen, Zsolt Illes

**Affiliations:** ^1^Department of Neurology, Odense University Hospital, Odense, Denmark; ^2^Department of Clinical Research, BRIDGE, University of Southern Denmark, Odense, Denmark; ^3^Department of Biochemistry and Molecular Biology, University of Southern Denmark, Odense, Denmark; ^4^Clinical and Experimental Multiple Sclerosis Research Center, Charité – Corporate Member of Freie Universität Berlin, Humboldt-Universität zu Berlin, and Berlin Institute of Health, Berlin, Germany; ^5^Department of Neurology, Charité – Corporate Member of Freie Universität Berlin, Humboldt-Universität zu Berlin, and Berlin Institute of Health, Berlin, Germany; ^6^Laboratory of Molecular Medicine, Department of Clinical Immunology, Sect. 7631, Rigshospitalet, Faculty of Health and Medical Sciences, University of Copenhagen, Copenhagen, Denmark; ^7^Institute of Neurology, Medical University of Vienna, Vienna, Austria; ^8^Department of Pathology, Odense University Hospital, Odense, Denmark; ^9^Department of Neurology, Rigshospitalet, Copenhagen University Hospital, Copenhagen, Denmark; ^10^Institute of Molecular Medicine, University of Southern Denmark, Odense, Denmark; ^11^Department of Neurology, Zealand University Hospital, Roskilde, Denmark; ^12^Neurobiology Research, Institute of Molecular Medicine, University of Southern Denmark, Odense, Denmark; ^13^NeuroCure Clinical Research Center, Charité – Corporate Member of Freie Universität Berlin, Humboldt-Universität zu Berlin, and Berlin Institute of Health, Berlin, Germany; ^14^Experimental and Clinical Research Center, Charite – Corporate Member of Freie Universität Berlin, Humboldt-Universität zu Berlin, and Berlin Institute of Health, Berlin, Germany; ^15^Center for Brain Research, Medical University of Vienna, Vienna, Austria

**Keywords:** CLIPPERS, proteomics, complement, cerebrospinal fluid, VCAM-1, ICAM-1, interleukin-8, multiple sclerosis

## Abstract

**Objective:**

Chronic lymphocytic inflammation with pontine perivascular enhancement responsive to steroids (CLIPPERS) is a rare syndrome with relapsing brainstem/cerebellar symptoms. To examine the pathogenic processes and investigate potential biomarkers, we analyzed combined materials of brain and cerebrospinal fluid (CSF) by comprehensive methodologies.

**Materials and methods:**

To identify major pathways of perivascular inflammation in CLIPPERS, we first compared the CSF proteome (*n* = 5) to a neurodegenerative condition, Alzheimer’s disease (AD, *n* = 5). Activation of complement was confirmed by immunohistochemistry (IHC) on CLIPPERS brain samples (*n* = 3) and by ELISA in the CSF. For potential biomarkers, we used biomarker arrays, and compared inflammatory and vessel-associated proteins in the CSF of CLIPPERS (*n* = 5) with another inflammatory relapsing CNS disease, multiple sclerosis (RMS, *n* = 9) and healthy subjects (HS, *n* = 7).

**Results:**

Two hundred and seven proteins in the CSF discriminated CLIPPERS from AD. The complement cascade, immunoglobulins, and matrix proteins were among the most frequently represented pathways. Pathway analysis of upstream regulators suggested the importance of vascular cell adhesion protein 1 (VCAM1), IFN-γ, interleukin (IL)-1, and IL-10. Differential regulation of more than 10 complement proteins of the 3 complement pathways in the CSF pointed to the role of complement activation. IHC on brain samples confirmed the perivascular complement activation, i.e., deposition of C3bc, C3d, and the terminal C5b-9 complement complex that partially overlapped with accumulation of IgG in the vessel wall. Besides endothelial cell damage, reactivity to smooth muscle actin was lost in the walls of inflamed vessels, but the glia limitans was preserved. The semi-quantitative array indicated that increased level of IL-8/CXCL8 (*p* < 0.05), eotaxin/CCL11 (*p* < 0.01), and granulocyte colony-stimulating factor (*p* < 0.05) in CSF could distinguish CLIPPERS from HS. The quantitative array confirmed elevated concentration of IL-8/CXCL8 and eotaxin/CCL11 compared to HS (*p* < 0.05, respectively) besides increased levels of ICAM-1 (*p* < 0.05) and VCAM-1 (*p* < 0.001). The increased concentration of VCAM-1 were able to differentiate CLIPPERS from RMS (*p* < 0.01), and a trend of elevated levels of ICAM-1 and IL-8/CXCL8 compared to RMS was also observed (*p* = 0.06, respectively).

**Conclusion:**

Complement activation, IgG deposition, and alterations of the extracellular matrix may contribute to inflammation in CLIPPERS. VCAM1, ICAM1, and IL-8 in the CSF may differentiate CLIPPERS from RMS.

## Introduction

Chronic lymphocytic inflammation with pontine perivascular enhancement responsive to steroids (CLIPPERS) is a rare relapsing disorder with subacute brainstem features, brain MRI displaying multiple punctate or curvilinear foci of gadolinium enhancement, and a clear radiological/clinical response to steroid treatment (1–3). Several cases have been described, which suggest that the typical MRI appearance can be seen in a variety of other disorders, including primary angiitis of the CNS, multiple sclerosis (MS), and lymphoma (4–8). Nevertheless, a homogenous group of patients has the classical features of CLIPPERS, and these patients do not develop other conditions even after a long observation period. These patients require long-term immunosuppression to prevent relapses, indicating that immune-mediated CNS inflammation is a key component (3).

The pathogenic mechanisms underlying the perivascular inflammation in CLIPPERS are largely unknown. Biopsies from affected areas show a predominant perivascular infiltration of CD3^+^ T cells, most of which are also CD4^+^ (1). This inflammation also expands to supratentorial brain regions that appear normal on 3 T MRI (1, 2, 9). CD68^+^ histiocytes can be present in moderate numbers, and infiltrating macrophages as well as a small number of neutrophils and eosinophils are found in some cases (2, 3, 10). B cells are generally seen in smaller numbers than T cells (1, 10). Some patients have transient or persistent oligoclonal bands (OCB) in the cerebrospinal fluid (CSF), suggesting that antibodies may also be of importance (1, 2, 9, 11, 12). The role of B cells in the pathogenesis may be also indicated by cases successfully treated with anti-CD20 (rituximab), a B cell depletion therapy to treat antibody-mediated diseases (3, 13).

Novel diagnostic criteria have recently been proposed based on evaluation of clinical features, MRI appearance and pathological examination of patients with suspected CLIPPERS. These criteria allow the diagnosis of definite CLIPPERS only after neuropathological examination and the diagnosis of possible CLIPPERS in patients with classical symptoms and MRI appearance but without available neuropathology. These criteria might be useful to discriminate true CLIPPERS from the many mimics described (10).

In this study, we aimed at identifying possible pathogenic mechanisms in CLIPPERS using patients fulfilling the new diagnostic criteria (10). First, we examined CSF samples by proteomics, and compared pathway regulations to Alzheimer’s disease (AD), a neurodegenerative disorder of the CNS with limited inflammation in order to get an overview of activated inflammatory pathways in CLIPPERS. We verified major findings by other assays and on brain tissue from patients. To evaluate proteomics data as possible biomarkers, we next compared inflammatory and vessel-associated proteins by arrays in CLIPPERS to healthy controls, and then to another relapsing CNS disease, multiple sclerosis (RMS).

## Materials and Methods

### Patients and Controls

Brain biopsy, CSF, and serum samples were obtained for diagnostic purposes. Pathological samples were retrospectively analyzed (Table 1). Patients #1–2 and #4–5 fulfilled the 2017 proposed diagnostic criteria for definite CLIPPERS (10). In patient #3a/b no biopsy was available, but the patient fulfilled the criteria for probable CLIPPERS (10).

**Table 1:** Demographics and methods used for the analysis of body fluids and tissues obtained from patients with chronic lymphocytic inflammation with pontine perivascular enhancement responsive to steroids.

	Patient 1 Danish	Patient 2 Danish	Patient 3a/b Danish^a^	Patient 4 German	Patient 5 Danish^b^
**Demographics and clinical data**					
Sex/age	F/58	M/42	M/60	F/69	M/62
Diagnosis	2007	2010	2013	2010	2008
Symptoms	Ataxia, dysarthria, paresthesia	Tetraspaticity, paraparesis, dysarthria, diplopia, ataxia	Diplopia, dysarthria, ataxia	Diplopia, dysarthria, dysphagia, ataxia, paresthesia	Diplopia, dysarthria, dysphagia, ataxia
Treatment	Prednisone, azathioprine	Prednisone, azathioprine	Prednisone, azathioprine	Methyl-prednisolone	Prednisone
Time of CSF collection	2014	2014	At diagnosis	At diagnosis	N/A
CSF, OCB	None	Persistent	None	None	None
Published	Ref. (11)	Ref. (11)	N/A	Ref. (9, 14)	Ref. (9)

**Cerebrospinal fluid**					
LC–MS/MS^c^	+	+	+/+	+	–
C3bs, TCC	+	+	+/+		
RayBiotech biomarkers	+	+	+/+	–	–
Mesoscale biomarkers	+	+	+/+	+	–
**Brain**					
C9neo	+	+	–	–	+
IgG	–	–	–	–	+
C3bc, C3d, GFAP, CD31, SMA	–	–	–	–	+

*^a^Same patient with sampling at two time points*.

*^b^Patient deceased*.

*^c^Non-modified proteins and posttranslational modifications*.

*CSF, cerebrospinal fluid; OCB, oligoclonal bands, LC–MS/MS; liquid chromatography tandem mass spectrometry; IHC, immunohistochemistry; TCC, soluble terminal complement complex; SMA, smooth muscle actin*.

#### Patients #1 and #2 with CLIPPERS

The 58-year-old woman and 42-year-old man had a relapsing course responding to steroids and MRI features of CLIPPERS. The clinical and radiological features have been described previously (11).

#### Patient #3a/b with CLIPPERS

The 60-year-old male was admitted in 2012 because of subacute ataxia, diplopia, and dysarthria. MRI revealed punctate gadolinium enhancement in the pons and cerebellar peduncle. CSF obtained twice showed no OCB. Symptoms and MRI changes remitted after high-dose corticosteroid treatment. After a second corticosteroid-responsive relapse in early 2013, azathioprine was started. His clinical condition is stable with low-dose steroid (10 mg prednisone daily) and azathioprine (175 mg daily).

#### Patient #4 With CLIPPERS

The 69-year-old woman was diagnosed with CLIPPERS in 2010. The clinical findings have been described previously (9, 14).

#### Patient #5 With CLIPPERS

The male patient died at the age of 62 years; autopsy data have recently been published (9).

#### Control Subjects

For the examination of CSF proteome, samples from five patients with AD were included (15). For the examination of cytokines, CSF samples from nine patients with RMS (16) without immunotherapy and from seven subjects without CNS disease (chronic headache) were used.

#### Standard Protocol Approvals, Registrations, and Patient Consents

This study was carried out in accordance with the recommendations of Declaration of Helsinki with written informed consent from all subjects. All subjects gave written informed consent in accordance with the Declaration of Helsinki. The protocol was approved by the regional ethical committee and the Danish Data Protection Agency (S-20120066).

### Proteomics

#### Sample Preparation and Labeling

Cerebrospinal fluid samples (*n* = 5) from patients #1–4 were used. Amino acid composition was analyzed for an estimation of total protein content, and the samples were treated with protease and phosphatase inhibitors. A pooled sample from patients #1 and #3a served as sample CLIPPERS-1, and a pooled sample from patients #2, #3b, and #4 served as sample CLIPPERS-2. These samples were compared to pooled CSF samples from five patients with AD. Samples were ultracentrifuged, and the supernatant was alkylated and digested (17). Peptides from each pool were labeled with iTRAQ reagent. After pooling of the individual iTRAQ channels, the peptides were separated into (i) neutral glycosylated peptides; subjected to hydrophilic interaction liquid chromatography (HILIC) before liquid chromatography tandem mass spectrometry (LC–MS/MS); (ii) non-modified peptides; fractioned by high pH reversed phase separation into six fractions and each fraction subjected to HILIC before LC–MS/MS; and (iii) sialylated N-linked glycopeptides and phosphorylated peptides; bound to TiO_2_ beads and subsequently analyzed using LC–MS/MS (17, 18).

#### LC–MS/MS

Peptides from the various fractions were analyzed by a nano-Easy LC (Thermo Fisher Scientific) coupled with a Q-Exactive mass spectrometer (Thermo Fisher Scientific, Bremen, Germany). All peptide fractions were re-suspended in 0.1% formic acid (FA) and loaded onto a 2 cm 100 µm inner diameter pre-column using the nano-Easy LC. Peptides were eluted directly onto the analytical column using a gradient of 0–34% buffer B (90% Acetonitrile, 0.1% FA) over 30–90 min depending on the UV intensity of the individual HILIC fractions. All LC–MS/MS runs were performed using an analytical column of 20 cm × 75 µm inner diameter fused silica, packed with C18 material (Dr. Maisch, Ammerbuch-Entringen, Germany). Mass spectrometry was performed using higher energy collision fragmentation (HCD) fragmentation on a Q-Exactive instrument. MS settings: a full MS scan in the mass area of 400–1,800 Da was performed in the Orbitrap with a resolution of 70,000 FWHM and a target value of 1 × 10^6^ ions. For each full scan the 12 most intense ions (charge states 2–5) were selected for HCD fragmentation and the fragments were detected at a resolution of 17,500 FWHM. Threshold for ion selection was 1.0e4, the AGC target value 2.0e4, activation time was 0.1 ms, isolation window was 1.5 Da, and normalized collision energy was 29.

#### Database Search

The MS raw files were processed and searched in Mascot and SEQUEST through the Proteome Discoverer 2.1 software (Thermo Fisher Scientific) against the human Uniprot database using the following parameters: precursor mass tolerance of 10 ppm; MSMS mass tolerance of 0.05 Da; enzyme: trypsin and up to two missed cleavages were allowed. For all data iTRAQ and carbamidomethylation was selected as fixed modifications. For the deglycosylated peptides the database search were performed with variable deamidation on *N*. When obtaining the final list of regulated proteins, we only used the proteins with two or more peptides. For the identification, the searched data were filtered to a threshold of 1% FDR using Percolator.

### Neuropathological Examination

#### Vascular Pathology

Paraffin embedded brainstem biopsy materials from patients #1 and #2 and autopsy material from patients #5 were stained with C9neo (poyclonal) and hematoxylin–eosin (HE). For positive controls in the C9neo stainings, biopsy samples from patients with anti-AQP4 seropositive neuromyelitis optica spectrum disorder (NMOSD) were used. Pathological specimens from the autopsy material (patient #5) were stained for IgG-cig and IgG-smi (Ventana Medical Systems, AZ, USA), GFAP (Ab6 Thermo Fisher Scientific), CD31 (PECAM; DAKO, polyclonal), C3bc (bH6; Hycult), and C3d (DAKO, polyclonal).

#### Indirect Immunofluorescence

Twenty-micrometer cryostat sections from formalin-fixed adult Sprague–Dawley rat brains were blocked with 5% goat serum followed by overnight incubation with undiluted CSF from patients #1–3. Following a standard washing procedure (Tris buffered saline), potential IgG binding was visualized with a secondary FITC-conjugated goat-anti-human IgG antibody (1:200, Alexa Flour 488, Thermofisher Scientific). Sections were counterstained with 10 µM 4′,6-diamidine-phenylindole dihydrochloride (DAPI, SigmaAldrich) and coverslipped using ProLong gold antifade (Thermofisher Scientific).

#### Binding of Isolated Serum IgG to CLIPPERS Brain

Three-micrometer brain stem sections from patient #6 were demasked and blocked with fetal bovine serum and were incubated with undiluted serum IgG (400 µg/ml), from patients #1–3 isolated by protein A affinity chromatography, acidic elution, and dialysis against PBS. Following standard washing procedure, sections were incubated for 2 h with HRP-conjugated rabbit anti-human IgG antibody (1:200, DAKO P0214). After a final rinse, sections were developed using diaminobenzidine dehydrated and coverslipped using Depex mounting medium. Digital images were obtained using a Leica microscope (Leica 4000B LED, Leica Microsystems, Wetzlar, Germany) equipped with a Leica digital camera (Leica DFC420, Leica Microimaging).

### Complement Activation in the CSF

Activation products C3bc and soluble terminal complement complex (TCC) were examined in the CSF as described (19, 20).

### Soluble Biomarker Assays

#### Semi-Quantitative Assay

Cerebrospinal fluid samples from patients #1–4 and three control subjects were applied to the RayBiotech TM Human Inflammation Array C3 as described by the manufacturer.^1^ All samples were run in duplicate. Intensity of streptavidin binding was measured by CCD images of protein spots and subsequent measurement of grayscale intensity (range 0–256 AU) using NIH image analysis software.^2^

#### Quantitative Analysis

Cerebrospinal fluid samples used in the RayBiotech assay (patients #1–4) were also applied to MesoScale V-PLEX Human Biomarker 37-Plex Kit (Mesoscale Discovery, Rockville, MD, USA). We also included CSF samples from seven controls and nine patients with RRMS. All samples were run in duplicate.

### Statistics

The identified and quantified peptides were imported into the Perseus program^3^ for statistic validation and *t*-testing to identify regulated proteins. Regulated proteins were exported and used for pathway analysis using the Ingenuity Pathway Analysis program and the STRING program.^4^ Proteins were used for quantitative analysis if they contained two or more unique peptides. The two-sample test was based on *p*-value threshold on 0.05. Mesoscale data were evaluated using one-way ANOVA with multiple comparisons and Tukey correction.

## Results

### Proteome of the CSF Obtained From CLIPPERS Patients

**Figure 1:**
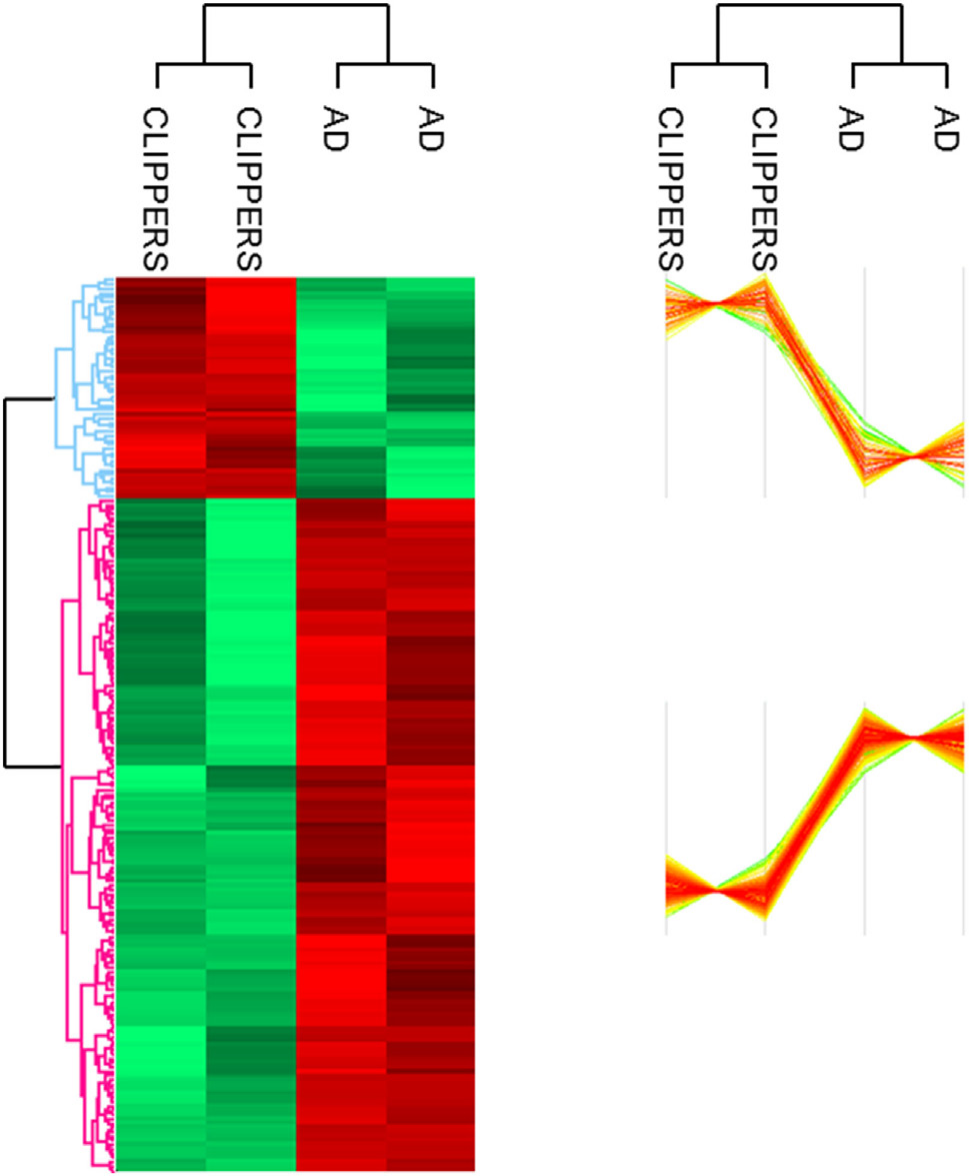
Proteomics of the cerebrospinal fluid in patients with chronic lymphocytic inflammation with pontine perivascular enhancement responsive to steroids (CLIPPERS). LC–MS/MS proteomics of the cerebrospinal fluid obtained from patients with CLIPPERS and Alzheimer disease was performed and compared as described in Section “Materials and Methods.” Heat maps of proteins discriminating between the two diseases are shown (red: upregulated, green: downregulated). The corresponding proteins are listed in Table S1 in Supplementary Material in the same order; upregulated proteins with functions are also indicated in Table 2.

Altogether, 207 proteins in the CSF proteome and PTMome could discriminate CLIPPERS from AD: 51 proteins were upregulated and 156 proteins were downregulated in the CSF of patients with CLIPPERS (Figure 1; Table 2; Table S1 in Supplementary Material).

**Table 2:** Upregulated proteins in the cerebrospinal fluid of patients with chronic lymphocytic inflammation with pontine perivascular enhancement responsive to steroids compared to patients with Alzheimer’s disease.

Protein	Function
**Endothelial cells**	
Isoform 2 of Ephrin type-A receptor 8	Receptor tyrosine kinase; cell adhesion and migration
Ephrin type-A receptor 4	Receptor tyrosin kinase; vascular formation and angiogenesis in the CNS; astrocyte differentiation; glial cell migration
Ephrin-B1	Cell surface ligand of receptor tyrosin kinases; cell–cell adhesion
Vascular cell adhesion protein 1 (CD106)	Cell adhesion and migration; ligand for VLA-4
Cell adhesion molecule 1	Cell–cell adhesion; NK cytotoxity; IFN-γ production by CD8^+^ T cells; risk factor for venous thrombosis
Beta-2-glycoprotein 1	Scavenges lipopolysaccharide; clears unwanted anionic cellular remnants; expressed on the surface and in endosomes of endothelial cells; target of antiphospholipid antibodies
Protocadherin-17 (PCDH17)	Cell–cell adhesion in the CNS; inhibits migration; promotes cell cycle arrest
Multimerin-1 (MMRN1, ECM)	Endothelial cell multimerin; carrier protein for platelet factors; ligand of integrins on activated platelets; coagulation and cell adhesion
Kallistatin	Tissue kallikrein inhibitor: vascular remodeling; inhibition of endothelial apoptosis; expressed by endothelial and smooth muscle cells of blood vessels

**Perivascular extracellular matrix**	
Vitronectin	Binds complement C9 and inhibits C9 polymerization and formation of the membrane attack complex (regulation of complement activation); plasminogen activation; negative regulation of blood coagulation; cell adhesion; its receptor is expressed on endothelial cells; arterial wall remodeling: promotes smooth muscle migration
Decorin	Inhibition of angiogenesis; binds to PDGF and inhibits its stimulatory activity on arterial smooth muscle cells
Laminin subunit gamma-1	Basement membrane assembly; cell adhesion and migration
Laminin subunit alpha-4	Regulates vascular endothelium cell survival; vessel wall formation in the skin
Extracellular matrix protein 2	ECM organization; heparin and integrin binding
Periostin	Predictor of airway eosinophilia [interleukin (IL)-4, IL-13]; induced by injury in smooth muscles of the neointima and adventitia; vascular remodeling in experimental allergic granulomatous angiitis
Multimerin-1	Extracellular matrix adhesion; adhesion of many different cell types *in vitro*, including activated platelets, neutrophils, and endothelial cells

**Serum complement**	
Complement C3	Complement activation; central role
Complement component C9	Complement activation; part of membrane attack complex (MAC)
Complement C2	Complement activation (classic and lectin pathways)
Complement C4-A	Derived from proteolysis of complement C4 (classic pathway): as anaphylatoxin induces local inflammation
C4b-binding protein beta chain	Complement inhibitor: controls the classic pathway
Complement factor I	Complement inhibitor: inactivates the complement components C4b and C3b
Plasma protease C1 inhibitor (SERPING1)	Complement inhibitor: controls activation of the C1 complex (classic pathway)
Vitronectin	Complement inhibitor: binds complement C9 and inhibits C9 polymerization and formation of the MAC

**Serum/plasma protein**	
Fibrinogen gamma chain	Coagulation
Prothrombin	Coagulation
Coagulation factor XI	Coagulation
Plasma kallikrein	Fibrinolysis, proteolysis, proinflammation and anti-angiogenesis
Alpha-2-antiplasmin	Inactivates plasmin and fibrinolysis, serine protease inhibitor
Plasma serine protease inhibitor	Inactivates serine proteases
Alpha-1-antichymotrypsin	Protease inhibitor
Alpha-1-acid glycoprotein 1 (orosomucoid)	Acute phase protein, anti-inflammatory role
Serum amyloid A-4 protein	Acute phase protein
Ig kappa chain V-III region VG (fragment)	Immunoglobulin (IgG)
IgG J chain	IgG
Ig delta chain C region	IgG
Ig lambda chain V-I region HA	IgG
Ig heavy chain V-III region 23	IgG
Insulin-like growth factor-binding protein 3 (IGFBP3)	Transport of IGF in the plasma
Inter-alpha-trypsin inhibitor heavy chain H1	Carries hyaluronan in plasma; may stimulate phagocytotic cells
Phospholipid transfer protein	Phospholipid transfer
Haptoglobin	Binds hemoglobin
Selenoprotein P	Secreted glycoprotein, selenium homeostasis
Phosphatidylethanolamine-binding protein 4	Secreted protein, lysosome, extracellular exosome; promotes cellular resistance to TNF-induced apoptosis

**Perivascular infiltrate and ubiquitous**	
V-type proton ATPase subunit S1	Ubiquitous, ion transmembrane transport
Macrophage colony-stimulating factor 1 receptor	Release of pro-inflammatory cytokines by macrophages
Receptor-type tyrosine-protein phosphatase gamma	Cell growth, differentiation
Cysteine-rich secretory protein 3	Neutrophil degranulation
L-selectin (CD62L)	Lymphocyte homing receptor
Ephrin-B1	T cell costimulation: negative feedback to TCR signals; neutrophils, macrophages and monocytes
Polypeptide *N*-acetylgalactosaminyltransferase 5	Protein glycosylation
Protocadherin fat 2	Membrane protein, cell adhesion

**Figure 2:**
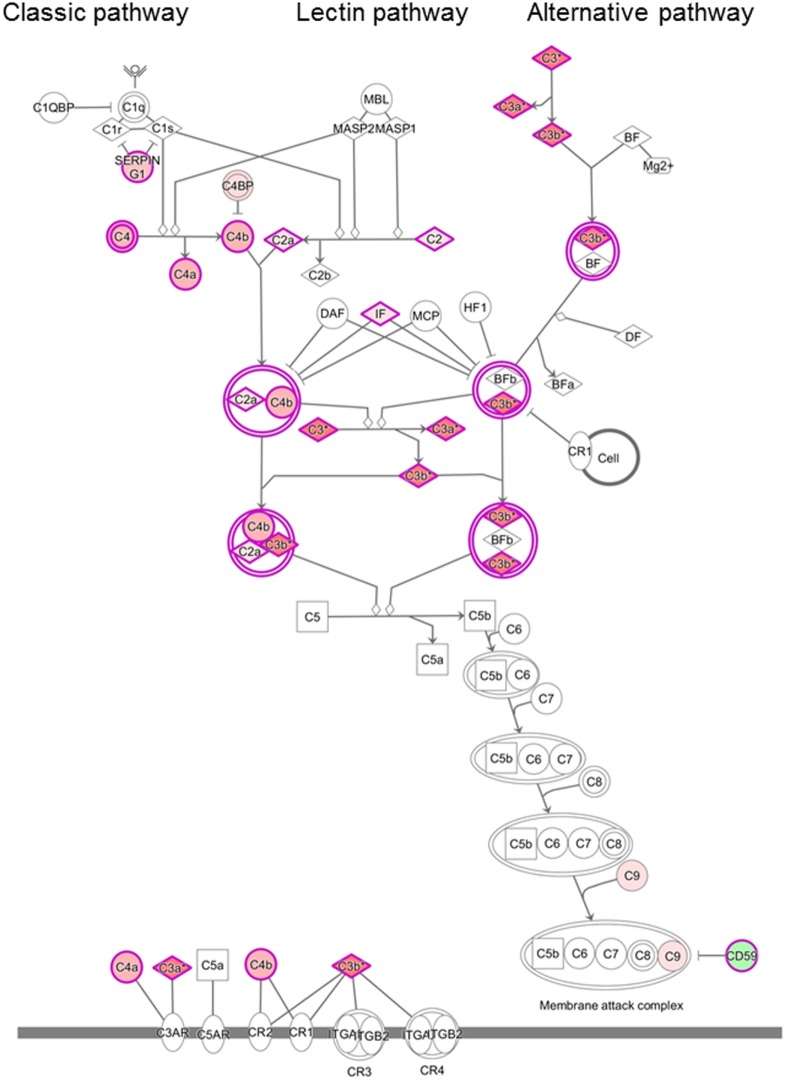
Differentially regulated complement pathways in the cerebrospinal fluid (CSF) of patients with chronic lymphocytic inflammation with pontine perivascular enhancement responsive to steroids (CLIPPERS). Ingenuity Pathway Analysis of the complement system in the CSF of patients with CLIPPERS. Red color indicates upregulation, green color indicates downregulation (see also Table 2 and Tables S1–S3 in Supplementary Material).

Analysis of the proteomics dataset revealed that the complement and coagulation cascades, as well as matrix proteins- and endothelium-related molecules, were among the most frequent pathways (Figure 2; Table 2; Figure S1 and Table S2 in Supplementary Material). Among the 12 top networks, molecules located in plasma membrane were overrepresented. Besides the complement proteins and immunoglobulins (IgGs), the network analysis also suggested the importance of vascular cell adhesion protein 1 (VCAM1), cytokines IFN-γ, interleukin (IL)-1, and IL-10 (Figure S1 in Supplementary Material; Table 2), and these cytokines were also identified as upstream regulators of several differentially regulated proteins (Table S3 in Supplementary Material).

To gather further supporting evidence that complement activation contributes to the pathogenesis of CLIPPERS syndrome, we next investigated complement activation products in the CSF and in brain biopsy/autopsy samples of patients with CLIPPERS.

### Complement Activation in the CLIPPERS CSF

Proteomics analysis of the CSF indicated that more than 10 proteins in the complement pathways were differentially regulated (Figure 2). To validate complement activation, we examined activation products in the CSF. The level of activation product C3bc and the soluble terminal complement complex (TCC) was measured in four CSF samples of three patients (#1, #2, #3a/b). In the CSF samples of patient #3a/b, concentration of C3bc was elevated (11.95 and 10.98 AU/ml; normal range 3–9 AU/ml), and soluble TCC was increased (0.184 and 0.117; normal range 0–0.013 AU/ml).

### Complement Activation in the CLIPPERS Brain

**Figure 3:**
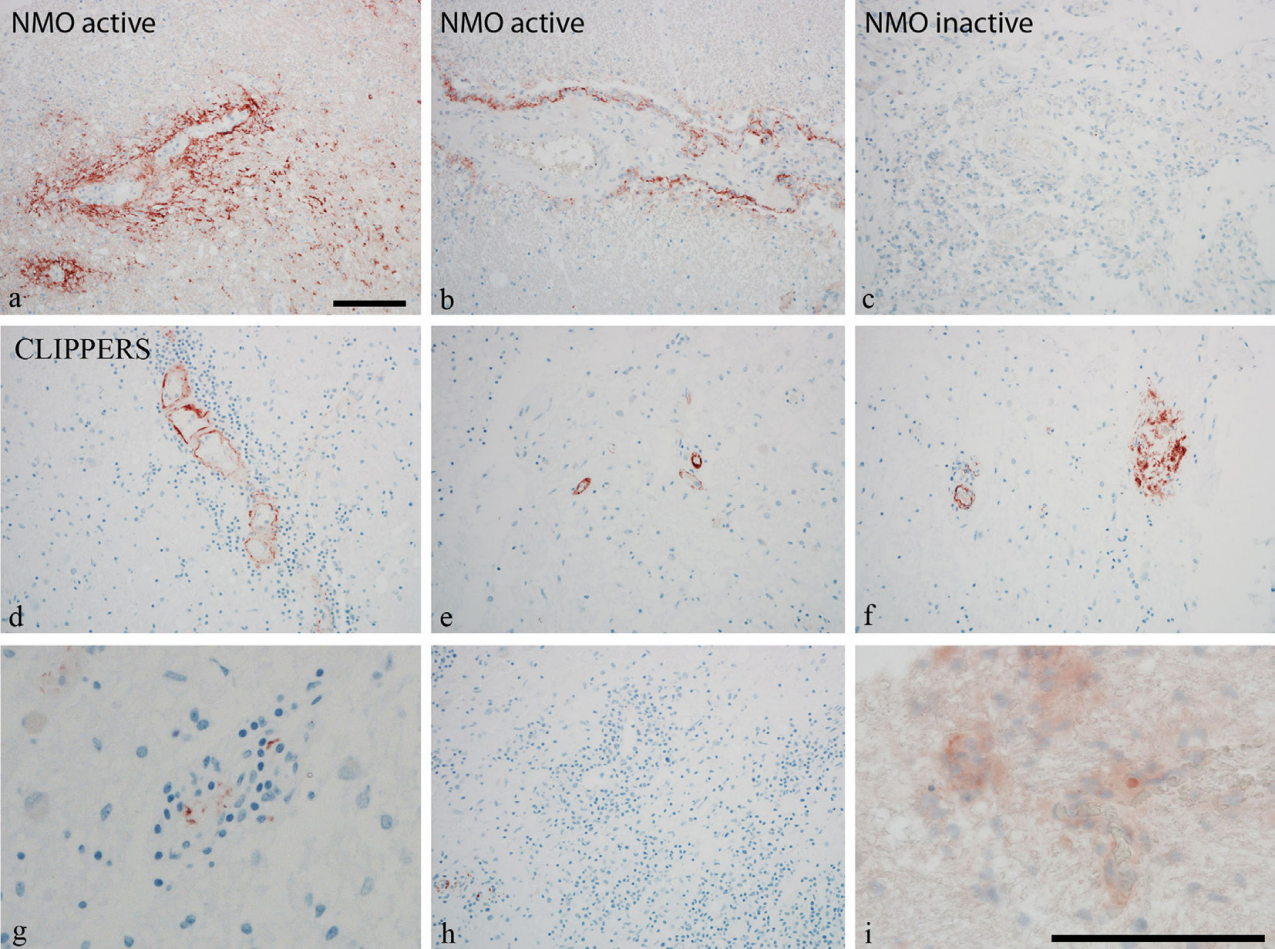
Perivascular complement deposition in the brain of patients with chronic lymphocytic inflammation with pontine perivascular enhancement responsive to steroids (CLIPPERS). Autopsy and biopsy samples from patients #1–2 and #5 were analyzed (see also Table 1). **(A–C)** Membrane attack complex (MAC) C9neo (red) reactivity in positive controls from patients with active and inactive neuromyelitis optica spectrum disorder (NMOSD). **(D–H)** MAC C9neo reactivity in autopsy (patient #5). **(I)** C9neo reactivity in biopsy specimen (patient #2). Sections were counterstained with toluidine blue. Magnification bars represent 100 μm.

To further validate the role of activated complement cascades in the pathogenesis of CLIPPERS, we examined deposition of the membrane attack complex (MAC) by the C9neo antibody on brain biopsy samples from two patients (#1, #2) and autopsy material from another patient (#5) (Figure 3). Proteomics of the CSF have been also analyzed in two of these three cases with brain materials (#1, #2, Table 1). Biopsy samples from patients with anti-AQP4 seropositive NMOSD served as controls (Figures 3A–C). We found C9neo staining in all three CLIPPERS patients. In the autopsy case, C9neo was found selectively in the vessel wall (Figures 3D–H) mainly with little inflammation, likely representing very early stages (Figures 3D,E). C9neo reactivity was also found in vessels, in which the wall was largely destroyed (Figure 3F). Less C9neo reactivity was seen in vessels with increasing inflammation, possibly representing late stages (Figures 3G–I).

### Vascular Pathology and Perivascular IgG Deposition

**Figure 4:**
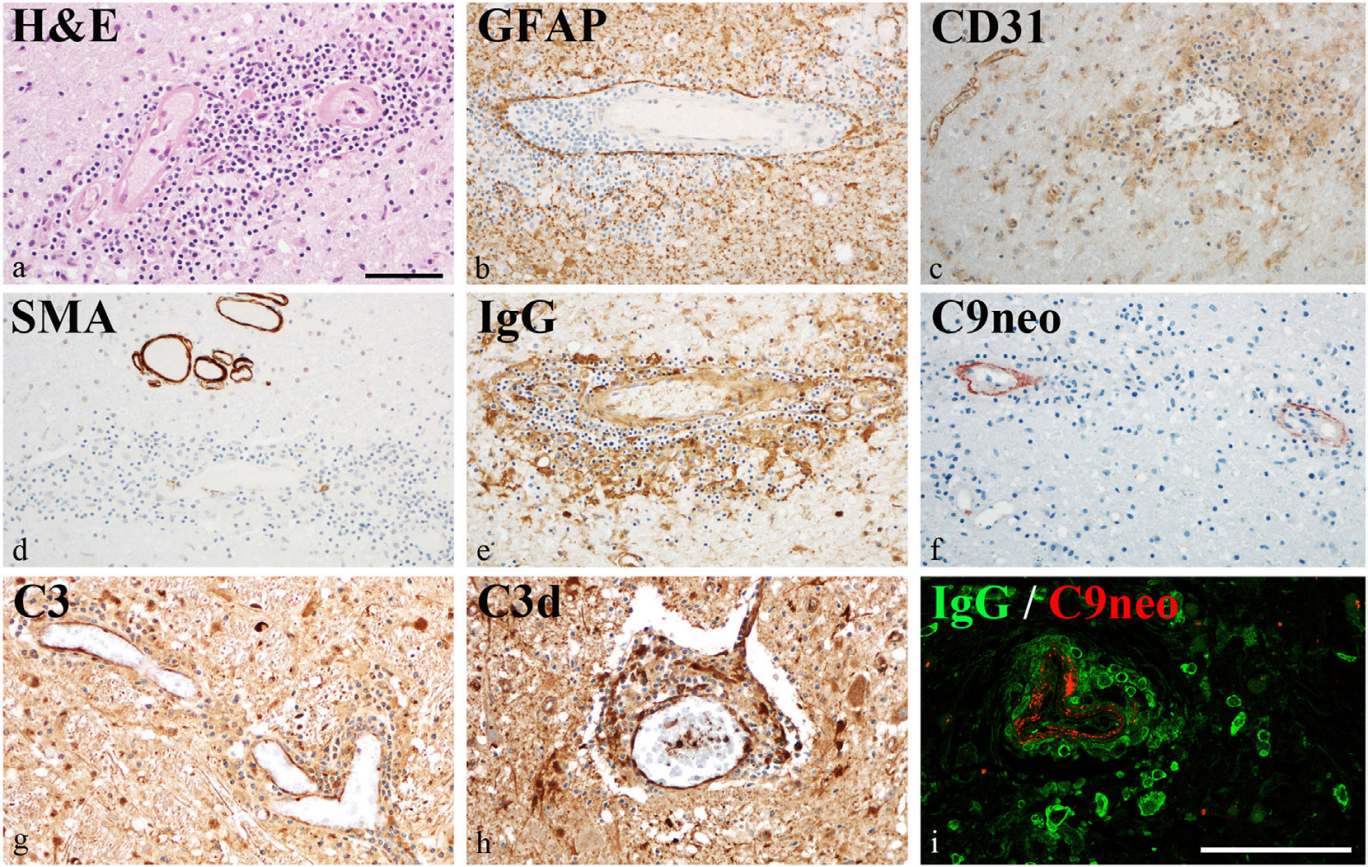
Vascular pathology in chronic lymphocytic inflammation with pontine perivascular enhancement responsive to steroids. Immunohistochemical analysis of brain samples of the autopsy case (patient #5). **(A)** Damage of the vessel wall is indicated by staining with hematoxylin–eosin. **(B)** Glia limitans is preserved (GFAP staining). **(C)** Partial loss of CD31 (PECAM) reactivity from endothelia of affected vessels indicates damage of endothelial cells. **(D)** Reactivity with smooth muscle actin (SMA) staining is lost in the walls of inflamed vessels. **(E)** This vessel pathology is associated with immunoglobulin (IgG) reactivity. **(F–H)** Complement activation (C3bc, C3d, and C9neo) is accentuated in the vessel wall. **(I)** IgG reactivity and complement activation partially overlap. Magnification bars represent 100 μm.

Damage of the vessel wall was indicated by homogeneous diffuse red staining with HE in autopsy material from patient #5 (Figure 4A). GFAP staining did not show major injury of astrocytes, and glia limitans was preserved (Figure 4B). By contrast, partial loss of CD31 (PECAM) reactivity of affected vessels indicated damage of endothelial cells (Figure 4C). The muscular vessel wall was also affected: smooth muscle actin (SMA) staining was in part lost in the walls of inflamed vessels (Figure 4D). This vessel pathology was associated with IgG deposition (Figures 4E,I) and complement activation (C3bc, C3d, and C9neo), which was clearly accentuated in the vessel wall (Figures 4F–I). Reactivity with IgG and complement deposition partially overlapped (Figure 4I).

To investigate specific IgG binding to CNS structures, we incubated a pool of four CSF samples from patients #1, #2, and #3a/3b with rat brain tissue. Isolated serum IgG from the same three patients were also incubated with formalin-fixed, paraffin-embedded tissue obtained from patient #5. We could not identify specific IgG staining in any of these experiments (data not shown).

### Potential Biomarkers of in the CSF of CLIPPERS

The obtained proteomics data suggested the role of cytokines and integrins in network, pathway, and upstream regulator analyses, and we also considered endothelial stress/activation based on the pathology data. Therefore, we next investigated if these molecules may differentiate CLIPPERS from healthy controls and another relapsing inflammatory disease, multiple sclerosis (RMS).

**Figure 5:**
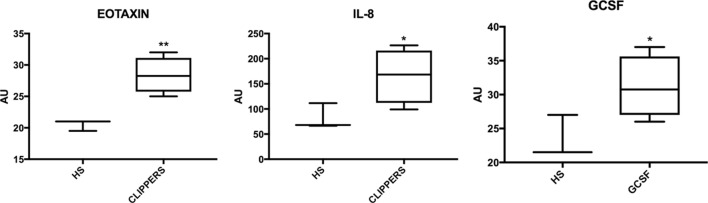
Molecular markers in the cerebrospinal fluid of patients with chronic lymphocytic inflammation with pontine perivascular enhancement responsive to steroids (CLIPPERS) compared to healthy subjects. Box-plots of molecular markers significantly upregulated in CLIPPERS CFS from patients #1–4 compared to healthy subjects (HS; *n* = 3) using the semi-quantitative RayBiotech human inflammatory assay. *p < 0.05; **p < 0.01 using Student’s *t*-test.

We first compared the CSF from CLIPPERS patients to healthy controls by the semi-quantitative RayBiotech array. IL-8, eotaxin (CCL11), and granulocyte colony-stimulating factor (GCSF) could distinguish CLIPPERS from HS (Figure 5).

**Figure 6:**
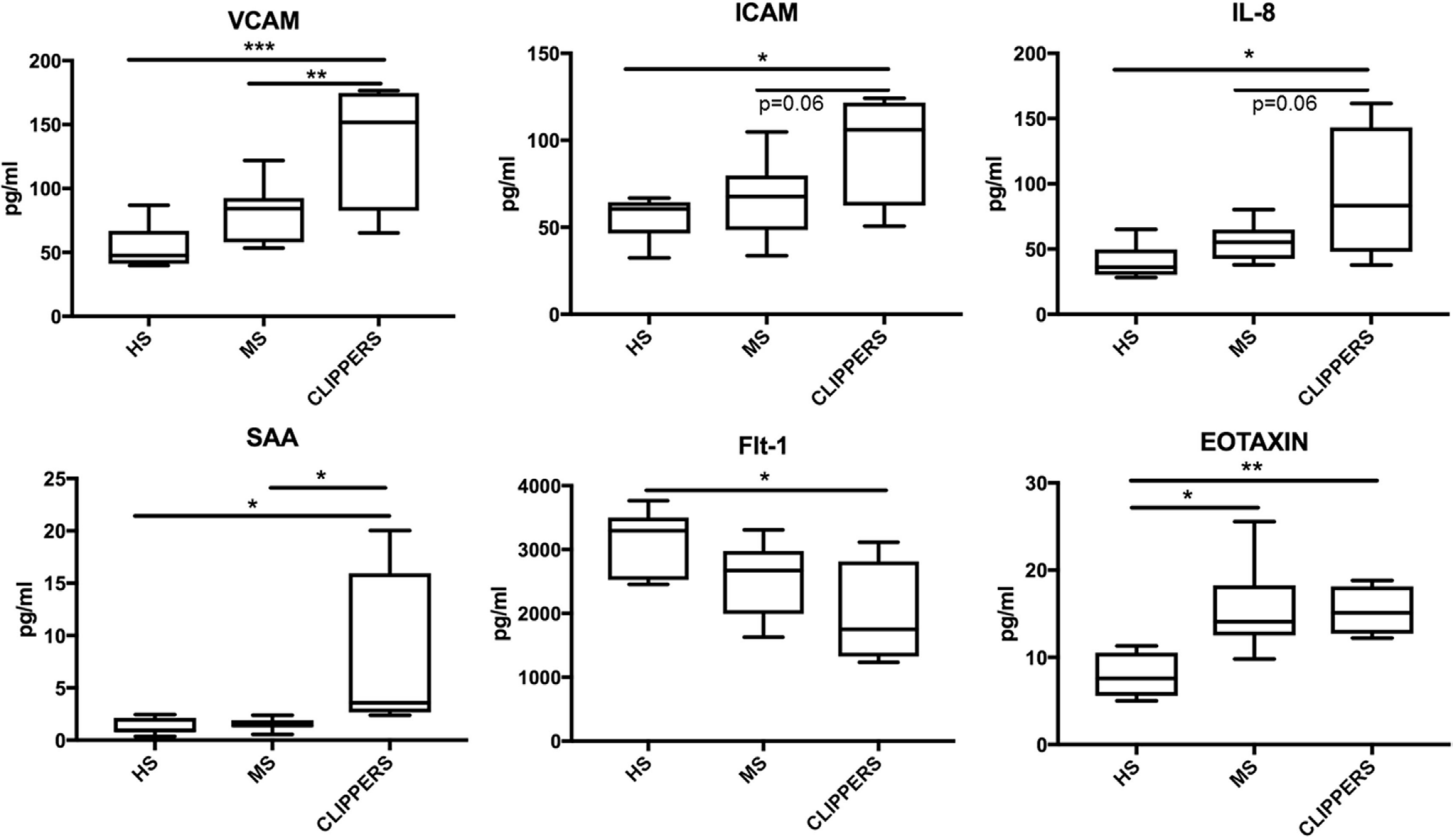
Molecular markers in the cerebrospinal fluid (CSF) of patients with chronic lymphocytic inflammation with pontine perivascular enhancement responsive to steroids (CLIPPERS) compared to patients with relapsing MS and healthy subjects. Quantitative Mesoscale V-PLEX 37 human assay: differentially expressed biomarkers in the CSF samples from patients #1–4, compared to relapsing-remitting multiple sclerosis (RMS; *n* = 9) and healthy subjects (HS; *n* = 7). Error bars indicate SD. *p < 0.05; **p < 0.01, **p < 0.001, one-way ANOVA with Tukey multiple comparison test.

Next, we compared CSF of CLIPPERS to CSF obtained from HS and RMS by the quantitative MesoScale V-PLEX Kit (Figure 6). This confirmed that IL-8 and eotaxin (CCL11) are increased the CSF of patients compared to HS. In addition, VCAM-1, ICAM-1, serum amyloid A, and soluble fms-like tyrosine kinase-1 (Flt1/VEGFR-1) could also distinguish CLIPPERS from HS. Elevated concentration of VCAM-1 distinguished the CLIPPERS CSF from RMS, and we also observed a trend of elevated IL-8 and ICAM-1 in the CSF of CLIPPERS compared to RMS (*p* = 0.06, respectively).

## Discussion

Mechanisms underlying inflammation in CLIPPERS are largely unknown. To investigate these, we examined the proteome in the CSF of CLIPPERS patients compared to non-inflammatory brain disease AD. AD is a classic neurodegenerative disease of the CNS. Although the role of innate immune responses is well established, adaptive immune-system driven effects, mediated by T and B cells, appear at present to be far less important in AD (21). There is evidence of T cell infiltration into the brain in AD, nevertheless perivascular adaptive inflammatory responses are absent in contrast in CLIPPERS (22). In the first step, therefore we aimed to identify the broadest spectrum of dysregulated proteins comparing two CNS diseases with different pathogenesis and cellular responses, thus also aiming at dissecting proteins related to CNS tissue injury from dysregulated proteins associated with CLIPPERS-specific inflammation. The overrepresentation of dysregulated proteins related to complement activation in the proteome of CLIPPERS was then validated on brain samples. By using a broad screening approach, we next compared the CSF of patients from CLIPPERS to another relapsing inflammatory CNS disease, relapsing MS and to healthy controls in order to partially validate proteomics data and to identify potential biomarkers for CLIPPERS.

Cerebrospinal fluid proteins that were regulated differently in CLIPPERS compared to AD included the following: (i) proteins related to endothelial damage and activation including molecules involved in leukocyte recruitment (e.g., adhesion molecules, VCAM-1, cell adhesion molecule 1) and vascular formation/remodeling (ephrin signaling); (ii) serum proteins, which are expected to reach the brain, when endothelial cells are damaged (e.g., IgGs, complement, coagulation factors, acute phase proteins, etc.); (iii) molecules of the perivascular extracellular matrix important in vascular wall formation and remodeling, regulation of coagulation and complement activation, and cell adhesion (e.g., vitronectin, decorin, laminin, periostin, multimerin-1, and extracellular matrix protein 2); and (iv) molecules related to perivascular infiltrates (e.g., CSF1R). Pathway and network analysis also indicated the importance of coagulation, cell adhesion and migration, the complement cascade, ephrin signaling, extracellular matrix-surface interaction, and contactin-mediated cell surface interactions. A network analysis for up-stream regulators of the differentially expressed proteins indicated the importance of several pro- and anti-inflammatory cytokines: IFN-γ, IL-1β, TNF-β, IL-6, IL-4, and IL-10. The differential presence of IgGs in the CSF proteome was unrelated to the presence of OCB; only one patient had temporary OCB in the CSF, and the hierarchical clustering identified similarly significant presence of IgGs and complement in the CSF samples without OCB.

We found upregulation of several key members of the complement pathways in CLIPPERS CSF: C4, C4a, C4b, C2, and C2a (lectin and classic pathways); C3, C3a, and C3b (classic, lectin, and alternative pathways); and C9 (part of the terminal MAC). Complement activation products and the terminal complement complex (TCC) could be detected around plaques also in AD (23), but the massive dysregulation of complement proteins in the CLIPPERS CSF compared to AD suggested a role of the complement system very different from that in a classic neurodegenerative disease. Our data indicated activation of the complement system, where at least one pathway (the classical or lectin pathway) and potentially the alternative pathway may drive activation. Complement activation was verified by elevated levels of C3bc and the soluble TCC in the CSF, and by perivascular deposition of the terminal MAC in three brains. These data altogether suggest the central role of complement activation in the pathogenesis of CLIPPERS. We also found perivascular deposition of IgG in vessels with largely destroyed walls confirming the CSF proteome data. Endothelial cells and the muscular vessel wall were primarily injured.

The deposition of complement and IgG may be a consequence of vascular damage due to leakage into the perivascular area; however, the increased levels of soluble TCC in the CSF and perivascular deposition of the MAC in the brain rather suggest local activation. The proteome and pathological data indicated endothelial damage and activation, leakage of serum proteins, vascular inflammation, and fibrosis with dysregulated perivascular extracellular matrix proteins. This may suggest that CLIPPERS is a form of vasculitis. The absence of vessel wall necrosis, granulocyte, or eosinophil infiltration does not support necrotizing vasculitis. Activation of the complement argues against an entirely T cell mediated vasculitis. If complement activation in CLIPPERS were mediated by antibodies, our data would suggest an antigen in the perivascular extracellular matrix. Autoimmunity against laminins has been described in autoimmune diseases (24–29), pregnancy loss (30), and Chagas disease (31). Complement 3 deficiency prolongs survival of laminin-deficient mice (32). Antibodies against laminin are able to fix and activate complement (33, 34). The clinical response to anti-CD20 (rituximab) also suggests that antibodies may play a role at least in some of the patients (3, 13). Still, we were unable to detect specific binding of IgGs by incubating brain tissue with CLIPPERS CSF and serum IgG with. This could be explained by low abundance of a given autoantibody in CSF/purified IgG pool from serum; nevertheless, histology of CLIPPERS brains indicated incomplete overlap between IgG and C9neo reactivity. This may argue against complement activation by specific autoantibodies. Two additional scenarios may explain complement activation: primary deficiency/local inefficiency of complement-regulating proteins, or alteration of extracellular matrix proteins that induces complement activation. We found downregulation of CD59 that blocks aggregation of C9 and formation of the MAC. Mutations in CD59 result in recurrent brain infarctions and absent protein expression on brain endothelial cells (35). Proteolytic fragments or exposed neo-epitopes by altered composition of the ECM may also activate complement (36, 37). Decorin binds C1q, and suppresses C1q-induced IL-8 production by endothelial cells; biglycan binds mannose-binding lectin and inhibits activation of the lectin pathway (38). Vitronectin regulates the MAC formation (39). Since a number of ECM proteins were differentially regulated in the CLIPPERS CSF proteome, complement activation by the alterations of ECM can be a possibility: induced by altered ECM exposing neo-epitopes or by leaking Ig, or C3b deposition in the presence of deficient inhibition by complement-regulating ECM proteins or CD59.

Cytokines were upregulated in the CLIPPERS CSF proteome, or were among upstream regulators, including IFN-γ, IL-1β, and IL-10. VCAM-1 was one of the most upregulated proteins and was overrepresented in networks and pathways. Considering these data that reflect the role of cytokines and the possible role of endothelial stress/activation, we decided to use a broader array screening approach. First, we compared CSF levels of cytokines, chemokines, and biomarkers related to angiogenesis/endothelial stress to HS with a semi-quantitative assay, then we used a quantitative array to compare data also to RMS besides HS.

Upregulated VCAM-1 in the CSF could distinguish CLIPPERS from both HS and RMS. Increased concentration of ICAM-1 was also observed compared to HS and as a trend when compared to RMS. These are key molecules in adhesion of lymphocytes and monocytes. Pro-inflammatory cytokines, such as IL-8, increase expression of ICAM-1 and VCAM-1 by endothelial cells (40). Indeed, increased concentration of IL-8 (CXCL8) in the CSF also differentiated CLIPPERS from HS, and there was a trend of elevated levels compared to RMS. IL-8 is produced by endothelial and glial cells, and mediates chemotaxis and neutrophil effector functions; neuroprotective and neurotrophic functions of CXCL8 are also emerging (41). Another chemokine eotaxin (CCL11) was also able to separate CLIPPERS from HS and RMS. Based on these findings, we suggest that upregulation of key molecules in chemotaxis, such as VCAM-1, ICAM-1, IL-8 (CXCL8), and eotaxin (CCL11) may serve as biomarkers for differentiating CLIPPERS from RMS. Moreover, GCSF could also differentiate CLIPPERS from HS and its upregulation may be a protective mechanism in response to inflammation (42). The tyrosine kinase sFlt-1 (VEGFR-1) was downregulated compared to HS. This molecule binds free placental growth factor and vascular endothelial growth factor, suppressing the proangiogenic effects (43). Thus, downregulation of sFlt-1 in CSF may indicate a net proangiogenic function in CLIPPERS.

This study is limited by the restricted number of patients. However, the number of reported cases worldwide is below 70, and CSF and brain samples are extremely rare. Here, we were able to combine analysis of both CSF and brain material, and in two patients, we even examined paired brain-CSF samples. Confirmation of results on samples from different compartments by other additional experiments and combination of different methods/compartments validating the findings strengthens the validity of our findings.

In conclusion, our data strongly suggest that perivascular complement activation is involved in the pathogenesis of CLIPPERS. Proteomics and molecular profiling of the CSF by soluble arrays points to the importance of vessel dysfunction: disruption of the BBB, attraction and adhesion of immune cells to endothelial cells, and angiogenesis. Upregulated VCAM-1, ICAM-1, IL-8, and eotaxin in the CSF may be potential biomarkers in CLIPPERS; nevertheless, their differentiating role from RMS should be confirmed by a higher number of CSF samples. The cause of complement activation in CLIPPERRS is unclear: complement binding, alteration of the ECM, and search for specific antigens may be candidates for further studies.

## Ethics Statement

This study was carried out in accordance with the recommendations of Declaration of Helsinki with written informed consent from all subjects. All subjects gave written informed consent in accordance with the Declaration of Helsinki. The protocol was approved by the regional ethical committee and the Danish Data Protection Agency (S-20120066).

## Author Contributions

MB and ZI: design and conceptualization, clinical data, analysis and interpretation of the data, drafting the manuscript. AH: performing proteomics, analysis of data. LD: analysis and interpretation of proteomics data. KR, DK, TS, HN, and FP: clinical data and interpretation. PG: examination of complement in the CSF, analysis and interpretation of data, drafting part of the manuscript. RH: indirect immunohistochemistry, analysis and interpretation of data. BC: indirect immunohistochemistry, analysis and interpretation of data, pathology. SH: isolation of IgG. PJ: analysis and interpretation of proteomics data, performing RayBiotech assay. MM: biomarker analysis and interpretation of data. HL: histology, analysis and interpretation of data, drafting part of the manuscript. ML: analysis and interpretation of proteomics.

## Conflict of Interest Statement

MB reports grant from Lundbeck A/S outside of the submitted work. AH, LD, PG, RH, BK, DK, TS, SH, HN, PJ, and MM report no disclosures. KR reports grants from German Ministry of Education and Research (BMBF/KKNMS, Competence Network Multiple Sclerosis) during the conduct of the study; grants and personal fees from Novartis, personal fees from Bayer Healthcare, personal fees from Biogen Idec, grants and personal fees from Merck Serono, personal fees from Sanofi-Aventis/Genzyme, personal fees from Teva Pharmaceuticals, grants from Guthy Jackson Charitable Foundation, outside the submitted work. FP reports grants and personal fees from various pharmaceutical companies outside the submitted work. HL reports personal fees from Novartis, personal fees from Sanofi Aventis, personal fees from TEVA, personal fees from Roche outside the submitted work. ZI reports grants from Lundbeckfonden, grants from Scleroseforeningen during the conduct of the study; personal fees from Biogen Idec, grants and personal fees from Sanofi Genzyme, personal fees from Novartis, personal fees from Merck Serono outside the submitted work.

## Footnotes

^1^http://www.raybiotech.com/files/manual/Antibody-Array/AAH-INF-3.pdf.

^2^https://imagej.nih.gov/nih-image/.

^3^http://www.perseus-framework.org.

^4^http://string-db.org.
