# Supplementary material for: Omics-Based Approach Reveals Complement-Mediated Inflammation in Chronic Lymphocytic Inflammation With Pontine Perivascular Enhancement Responsive to Steroids (CLIPPERS)
Source: Front Immunol. 2018 Apr 23;9:741. doi: 10.3389/fimmu.2018.00741 (PMC5925867; doi:10.3389/fimmu.2018.00741)
Supplement: Supplementary file 1 [file Presentation_1.PDF]

Supplementary Figure 1A: Networks of differentially regulated proteins in the CSF of CLIPPERS

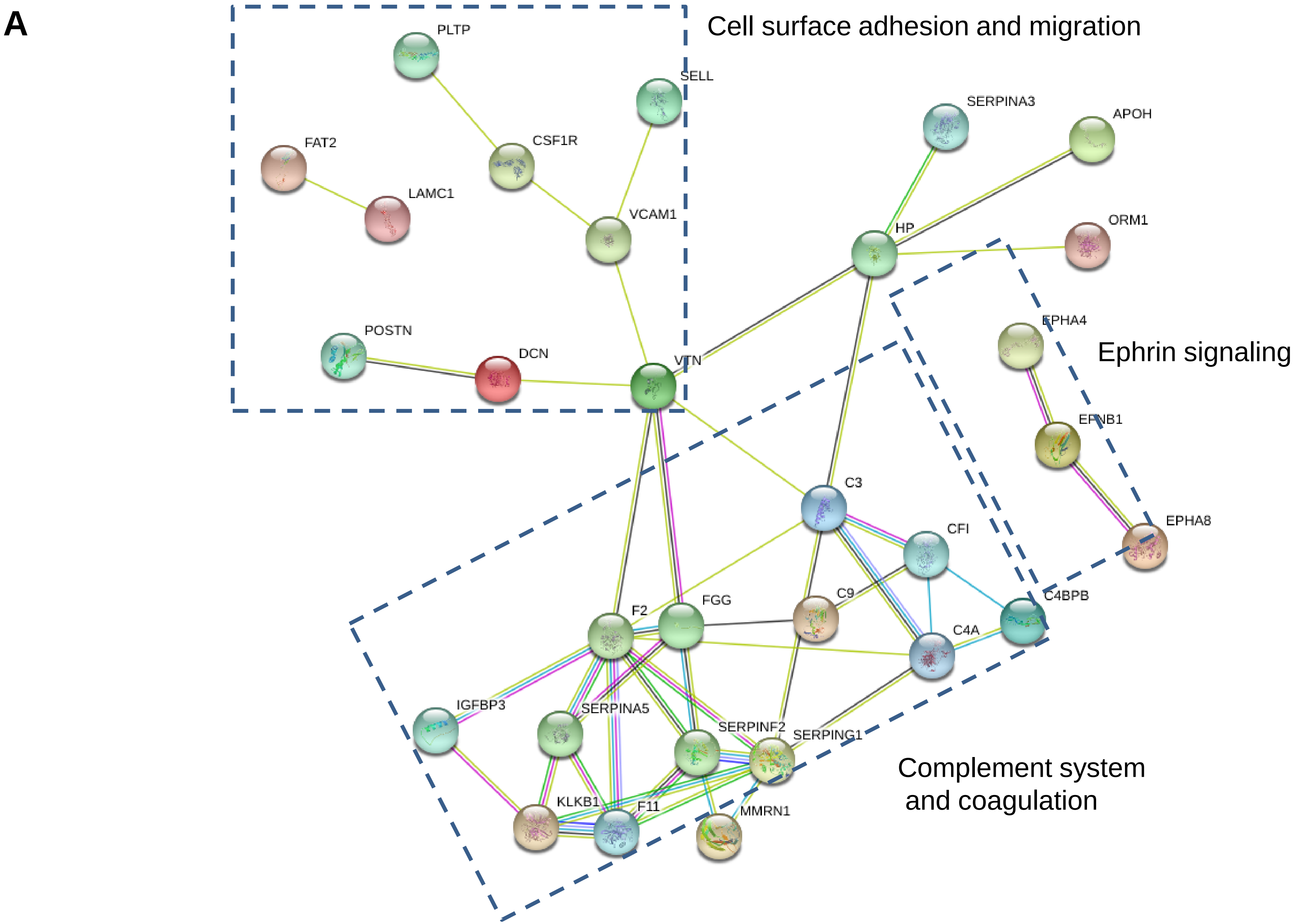

A. Network of cellular movement, cell-to-cell signaling and interaction, and inflammatory response of upregulated proteins by STRING program (see also Supplementary Table 1, 2 and 3).

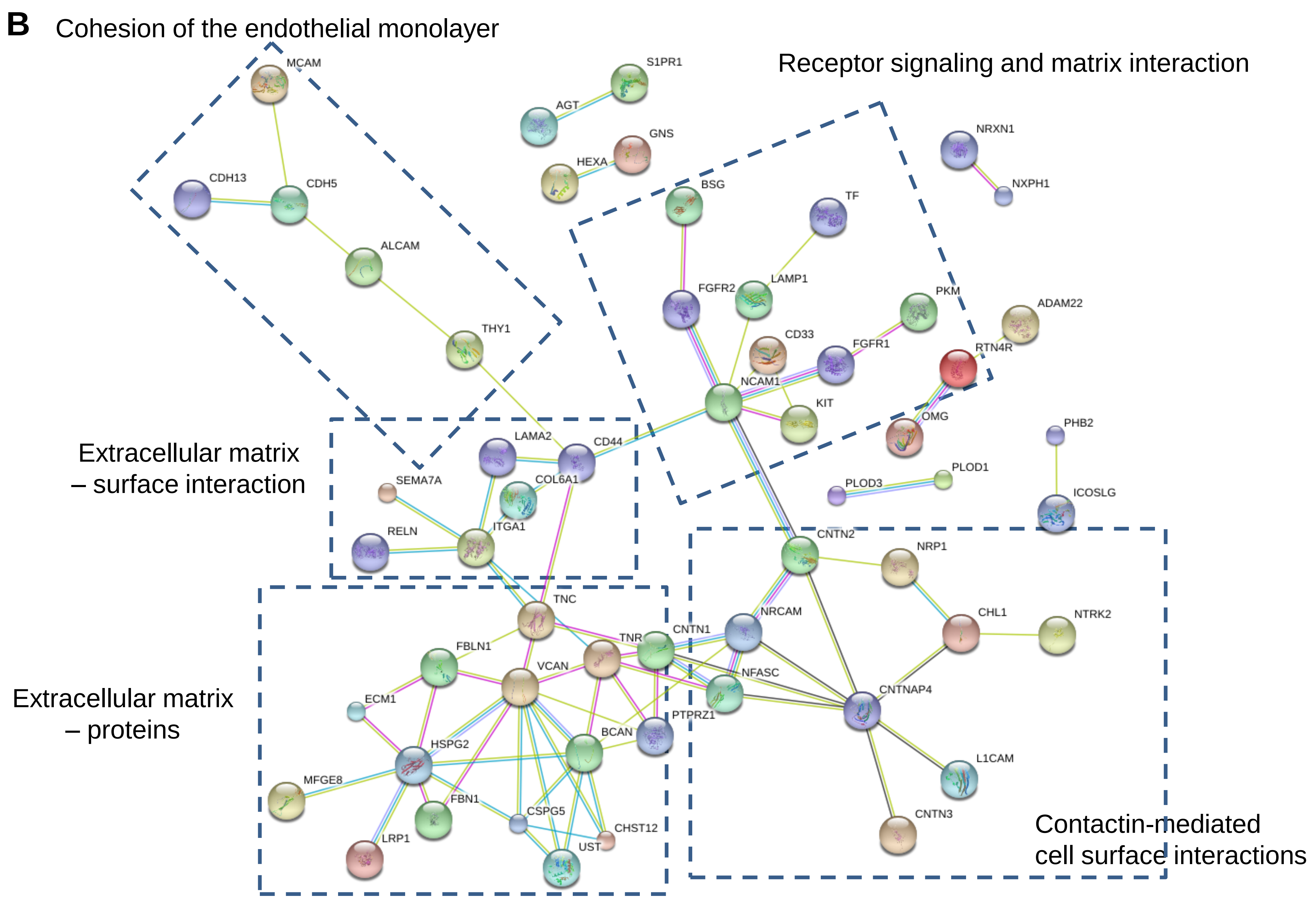

B. Merged networks of cellular movement, immune cell trafficking, hematological system development and function, cellular growth and proliferation, and tissue development of downregulated proteins by STRING program (*see also Supplementary Table 1, 2 and 3*).
