# Supplementary material for: Omics-Based Approach Reveals Complement-Mediated Inflammation in Chronic Lymphocytic Inflammation With Pontine Perivascular Enhancement Responsive to Steroids (CLIPPERS)
Source: Front Immunol. 2018 Apr 23;9:741. doi: 10.3389/fimmu.2018.00741 (PMC5925867; doi:10.3389/fimmu.2018.00741)
Supplement: Supplementary file 2 [file Table_1.PDF]

Supplementary Table 1

Differentially regulated proteins in the CSF of patients with CLIPPERS compared to patients wih Alzheimer disease

Differentially regulated proteins, CLIPPERS[a]	
Up-regulated	Down-regulated
Isoform 2 of Ephrin type-A receptor 8	Follistatin-related protein 4
Vascular cell adhesion protein 1	Polypeptide N-acetylgalactosaminyltransferase 18
Complement C3	Cadherin EGF LAG seven-pass G-type receptor 2
Vitronectin	Sphingosine 1-phosphate receptor 1
Decorin	Procollagen-lysine,2-oxoglutarate 5-dioxygenase 3
Fibrinogen gamma chain	Contactin-3
Cell adhesion molecule 1	Fibronectin type III domain-containing protein 5
Insulin-like growth factor-binding protein 3	Tenascin ECM
Ephrin type-A receptor 4	Sortilin-related receptor
Galectin-3-binding protein	Putative phospholipase B-like 2
Selenoprotein P	Afamin
Complement component C9	Opioid-binding protein/cell adhesion molecule
Alpha-1-antichymotrypsin	Neurexophilin-1
L-selectin	Cation-independent mannose-6-phosphate receptor
Complement factor I	Serum albumin
Alpha-1-acid glycoprotein 1	Leucine zipper protein 2
Plasma kallikrein	Heparan-sulfate 6-O-sulfotransferase 3
Immunoglobulin J chain	Mannosyl-oligosaccharide 1,2-alpha-mannosidase IA
V-type proton ATPase subunit S1	CD59 glycoprotein compl inhib
Macrophage colony-stimulating factor 1 receptor	Trophoblast glycoprotein
Prothrombin	Neural cell adhesion molecule 1
Plasma protease C1 inhibitor	GDP-fucose protein O-fucosyltransferase 2
Inter-alpha-trypsin inhibitor heavy chain H1	Isoform C of Fibulin-1 ECM
Phosphatidylethanolamine-binding protein 4	Oligodendrocyte-myelin glycoprotein
Complement C2	Procollagen-lysine,2-oxoglutarate 5-dioxygenase 1
Laminin subunit gamma-1	Prosaposin receptor GPR37
Extracellular matrix protein 2	Inactive phospholipase D5
Polypeptide N-acetylgalactosaminyltransferase 5	Prostate androgen-regulated mucin-like protein 1
Coagulation factor XI	Collagen alpha-1(VI) chain
Cysteine-rich secretory protein 3	SPARC-like protein 1
Beta-2-glycoprotein 1	CD166 antigen (activated leukocyte cell adhesion molecule, ALCAM)
Complement C3	Neuronal cell adhesion molecule
Receptor-type tyrosine-protein phosphatase gamma	Voltage-dependent calcium channel subunit alpha-2/delta-1
Complement C4-A	Neuroserpin
Ig kappa chain V-III region VG (Fragment)	Immunoglobulin superfamily containing leucine-rich repeat protein 2

Serum amyloid A-4 protein
Phospholipid transfer protein
Laminin subunit alpha-4
Protocadherin-17
Kallistatin
Multimerin-1
Ephrin-B1
Ig delta chain C region
Ig lambda chain V-I region HA
Plasma serine protease inhibitor
Alpha-2-antiplasmin
C4b-binding protein beta chain
Ig heavy chain V-III region 23
Periostin
Haptoglobin
Protocadherin Fat 2

Alpha-N-acetylglucosaminidase
Hepatocyte cell adhesion molecule
Isoform 2 of SPARC-related modular calcium-binding protein 1
Integral membrane protein DGCR2/IDD
Neuroblastoma suppressor of tumorigenicity 1
Thy-1 membrane glycoprotein
SLIT and NTRK-like protein 1
Neuropilin-1
Chondroitin sulfate proteoglycan 5
Neural cell adhesion molecule L1
Reticulon-4 receptor
Prosaposin receptor GPR37L1
Kallikrein-11
Fibroblast growth factor receptor 2
Carboxypeptidase Q
Disintegrin and metalloproteinase domain-containing protein 10
Kallikrein-6
Transmembrane protein 130
Zinc transporter ZIP6
Fibroblast growth factor receptor 1
Tenascin-R
Serotransferrin
Protocadherin-9
Cadherin-13
Lysosome-associated membrane glycoprotein 1
Voltage-dependent calcium channel subunit alpha-2/delta-2
Limbic system-associated membrane protein
Beta-Ala-His dipeptidase
Multiple epidermal growth factor-like domains protein 10
Bone morphogenetic protein receptor type-2
Fibrillin-1
Attractin
Uronyl 2-sulfotransferase
Fibroblast growth factor receptor 3
Phospholipase D3
Nectin-1
Anthrax toxin receptor 1
Clusterin-like protein 1
Contactin-1
Serotransferrin
Cell adhesion molecule 4
Netrin receptor UNC5A
N-acetylglucosamine-6-sulfatase
Twisted gastrulation protein homolog 1
Probable serine carboxypeptidase CPVL
Tyrosine-protein kinase receptor UFO

Osteopetrosis-associated transmembrane protein 1
CD44 antigen
Cadherin-5
Neural cell adhesion molecule 2
Prohibitin-2
Biotinidase
Receptor tyrosine-protein kinase erbB-3
Ribonuclease T2
Integrin alpha-1
BDNF/NT-3 growth factors receptor
Contactin-2
Sodium/iodide cotransporter
Myeloid cell surface antigen CD33
Dickkopf-related protein 3
Cell surface glycoprotein MUC18
Carbohydrate sulfotransferase 12
Protein kinase C-binding protein NELL2
Plexin-B3
Tumor necrosis factor receptor superfamily member 21
IgLON family member 5
Basement membrane-specific heparan sulfate proteoglycan core protein
MAM domain-containing glycosylphosphatidylinositol anchor protein 1
Tyrosine-protein phosphatase non-receptor type substrate 1
Secretogranin-1
Disintegrin and metalloproteinase domain-containing protein 22
Retinol-binding protein 4
Semaphorin-4D
Neurofascin
Prostaglandin-H2 D-isomerase
Serotransferrin
Cell adhesion molecule 4
Netrin receptor UNC5A
N-acetylglucosamine-6-sulfatase
Twisted gastrulation protein homolog 1
Probable serine carboxypeptidase CPVL
Tyrosine-protein kinase receptor UFO
Osteopetrosis-associated transmembrane protein 1
CD44 antigen
Cadherin-5
Neural cell adhesion molecule 2
Prohibitin-2
Biotinidase
Receptor tyrosine-protein kinase erbB-3
Ribonuclease T2
Integrin alpha-1

BDNF/NT-3 growth factors receptor
Contactin-2
Sodium/iodide cotransporter
Myeloid cell surface antigen CD33
Dickkopf-related protein 3
Cell surface glycoprotein MUC18
Carbohydrate sulfotransferase 12
Protein kinase C-binding protein NELL2
Plexin-B3
Tumor necrosis factor receptor superfamily member 21
IgLON family member 5
Basement membrane-specific heparan sulfate proteoglycan core protein
MAM domain-containing glycosylphosphatidylinositol anchor protein 1
Tyrosine-protein phosphatase non-receptor type substrate 1
Secretogranin-1
Disintegrin and metalloproteinase domain-containing protein 22
Retinol-binding protein 4
Semaphorin-4D
Neurofascin
Prostaglandin-H2 D-isomerase
Versican core protein
Prolow-density lipoprotein receptor-related protein 1
UPF0454 protein C12orf49
ICOS ligand
Interleukin-6 receptor subunit beta
Basigin
Brevican core protein
Ephrin-A3
Pyruvate kinase PKM
Semaphorin-4B
Lactadherin
Isoform 2 of Neural cell adhesion molecule L1-like protein
Transmembrane protease serine 5
Beta-hexosaminidase subunit alpha
Folate receptor beta
Protein CREG1
Cerebellin-2
Reelin
Ribonuclease pancreatic
Laminin subunit alpha-2
Mast/stem cell growth factor receptor Kit
Semaphorin-7A
Tetraspanin-3

	Seizure 6-like protein
	Membrane protein FAM174A
	Leucine-rich repeat-containing protein 4B
	Inactive serine protease PAMR1
	Pro-cathepsin H
	Carboxypeptidase E
	Prosaposin
	Tyrosine-protein kinase receptor Tie-1
	Neurexin-1
	Semaphorin-4C
	Multiple epidermal growth factor-like domains protein 8
	Isoform 2 of CD166 antigen
	Contactin-associated protein-like 4
	OX-2 membrane glycoprotein
	Cathepsin F
	Zinc-alpha-2-glycoprotein
	Extracellular matrix protein 1
	Delta and Notch-like epidermal growth factor-related receptor
	Angiotensinogen
	Carbonic anhydrase-related protein 11
	Receptor-type tyrosine-protein phosphatase zeta
	Receptor-type tyrosine-protein phosphatase kappa
	Uncharacterized protein KIAA1467

[a]The proteins are listed in the same order as presented in the heatmap of Figure 1.
