# Supplementary material for: Omics-Based Approach Reveals Complement-Mediated Inflammation in Chronic Lymphocytic Inflammation With Pontine Perivascular Enhancement Responsive to Steroids (CLIPPERS)
Source: Front Immunol. 2018 Apr 23;9:741. doi: 10.3389/fimmu.2018.00741 (PMC5925867; doi:10.3389/fimmu.2018.00741)
Supplement: Supplementary file 3 [file Table_2.PDF]

Supplementary Table 2

Differentially regulated pathways in the CSF of patients with CLIPPERS compared to patients with Alzheimer disease

Pathway ID	Pathway description	Count in gene set	False discovery rate
	Biological process		
up-regulated			
GO:0072376	protein activation cascade	11	1.33e-14
GO:0030195	negative regulation of blood coagulation	8	2.18e-10
GO:1903034	regulation of response to wounding	13	7.94e-10
GO:0070613	regulation of protein processing	8	3.88e-09
GO:0006952	defense response	19	6.18e-09
down-regulated			
GO:0007155	cell adhesion	46	2.36e-22
GO:0051960	regulation of nervous system development	33	2.89e-15
GO:0007399	nervous system development	51	5.23e-14
GO:0009653	anatomical structure morphogenesis	53	5.68e-14
GO:0022008	neurogenesis	43	5.68e-14
	Molecular function		
up-regulated			
GO:0004866	endopeptidase inhibitor activity	8	2.73e-06
GO:0005539	glycoseaminoglycan binding	8	6.42e-06
GO:0008201	heparin binding	7	1.56e-05
GO:0004867	serin-type endopeptidase inhibitor activity	6	2.37e-05
GO:0050839	cell adhesion molecule binding	5	0.00326
down-regulated			
GO:0019199	transmembrane receptor protein kinase activity	10	9.17e-07
GO:0004714	transmembrane receptor protein tyrosine kinase activity	9	1.48e-06
GO:0004872	receptor activity	31	1.23e-05
GO:0030246	carbohydrate binding	13	0.000126
GO:0004888	transmembrane signaling receptor	25	0.000221

	activity		
Cellular component			
up-regulated			
GO:0005615	extracellular space	30	5.81e-24
GO:0072562	blood microparticle	14	3.41e-19
GO:0070062	extracellular exosome	32	3.67e-17
GO:0044421	extracellular region part	34	5.11e-16
GO:0005576	extracellular region	35	7.35e-15
down-regulated			
GO:0005576	extracellular region	97	2.38e-28
GO:0031224	intrinsic component of membrane	96	4.97e-20
GO:0044421	extracellular region part	79	4.97e-20
GO:0070062	extracellular exosome	66	4.24e-18
GO:0005615	extracellular space	44	5.34e-17
KEGG pathways			
up-regulated			
04610	complement and coagulation cascade	11	4.73e-16
05150	S. aureus infection	3	0.0258
05133	Pertussis	3	0.0421
down-regulated			
04514	cell adhesion molecules	14	5.23e-10
04512	ECM-receptor interaction	8	3.46e-05
04142	lysosome	8	0.000291
04360	axon guidance	8	0.000291
04151	PI3K-Akt signaling pathway	10	0.0129
