# Supplementary material for: Omics-Based Approach Reveals Complement-Mediated Inflammation in Chronic Lymphocytic Inflammation With Pontine Perivascular Enhancement Responsive to Steroids (CLIPPERS)
Source: Front Immunol. 2018 Apr 23;9:741. doi: 10.3389/fimmu.2018.00741 (PMC5925867; doi:10.3389/fimmu.2018.00741)
Supplement: Supplementary file 4 [file Table_3.PDF]

Supplementary Table 3

Upstream regulators of differentially expressed proteins in the CSF of patients with CLIPPERS

Upstream regulator	Molecule type	P-value of overlap	Target molecules
IFNG	cytokine	0.0000197	AGT,BCAN,C2,C3,C4A/C4B,CD200,CD44,CELSR2,ICOSLG/LOC102723996,LAMC1,NTRK2,PCDH17,SEMA7A,SERPINA5,THY1, VCAM1
DGCR8	enzyme	0.0000514	ERBB3,NCAM1,NRCAM,SMOC1,TNC
HTT	transcription regulator	0.0000839	AGT,COL6A1,CSF1R,DCN,DKK3,MAN1A1 NTRK2,PLOD3,RBP4,SELENOP,SERPINA3, SERPING1,VCAM1
DIO2	enzyme	0.000102	CNTN2,COL6A1,ICOSLG/LOC102723996, SEMA7A
LRPAP1	other	0.000129	LRP1,SORL1
NPAS3	other	0.000129	FGFR1,RELN
MYOC	other	0.000482	LAMA2,LAMC1,NFASC
COL18A1	other	0.000863	ANTXR1,F11,FGFR1,NRP1,TNFRSF21
NFASC	other	0.000879	BCAN,NFASC,NRCAM
KIT	transmembrane receptor	0.00126	CD44,KIT
ADCYAP1	other	0.00146	CNTNAP4,FGFR2,MAN1A1,POSTN,SEZ6L
BMPR2	kinase	0.00188	EFNB1,EPHA4
PDPK1	kinase	0.00188	S1PR1,SELL
JUN	transcription regulator	0.00198	CHL1,IGFBP3,NRCAM,TNC
SLC16A2	transporter	0.00312	CNTN2,ICOSLG/LOC102723996,SEMA7A
TCIRG1	enzyme	0.00345	CD44,SELL
LAMA2	other	0.00441	CDH5,LAMA2
CD44	enzyme	0.00547	CDH5,SELL
NFKBIA	transcription regulator	0.00617	C3,PTPRZ1,VCAM1
APP	other	0.00719	AXL,BSG,C3,C4A/C4B,DCN,L1CAM,NRP1, PKM,RELN,VCAM1
XBP1	transcription regulator	0.0079	S1PR1,VCAM1
NR1H2	ligand-dependent nuclear receptor	0.00915	AGT,C3,PLTP
IL10RA	transmembrane receptor	0.00999	C3,DCN,ECM1,FBN1,FOLR2,POSTN,S1PR1 SELL
SLC9A6	transporter	0.0114	NTRK2
NPAS1	transcription regulator	0.0114	RELN
PHF6	other	0.0114	CSPG5
NEUROD6	transcription regulator	0.0114	CNTN2
RAB27A	enzyme	0.0114	CD44
GNAS	enzyme	0.0114	NRP1

SOS2	other	0.0114	SELL
TP73	transcription regulator	0.0114	RELN
MAG	other	0.0114	RTN4R
SOS1	other	0.0114	SELL
FHL2	transcription regulator	0.0114	S1PR1
MEOX2	transcription regulator	0.0114	LRP1
Ank2	other	0.0114	L1CAM
TOP2B	enzyme	0.0114	RELN
SH3BP2	other	0.0114	KIT
NFIB	transcription regulator	0.0114	EFNB1
ADCY3	enzyme	0.0114	NRP1
IL1B	cytokine	0.0137	C3,SERPINA3,SERPINF2,VCAM1
PPARA	ligand-dependent nuclear receptor	0.0139	ADAM10,VCAM1
VEGFA	growth factor	0.0145	CDH5,ITGA1,NRP1
S100A9	other	0.0171	GPR37L1,PLXNB3,POSTN,UNC5A,VCAN
S100A8	other	0.0176	GPR37L1,PLXNB3,POSTN,UNC5A,VCAN
TCF7L2	transcription regulator	0.0186	ERBB3,FGFR2,LAMP1,MAN1A1,MCAM, NFASC,SEMA4D
HMOX1	enzyme	0.0194	RELN,VCAM1
Cdc42	enzyme	0.0194	CTSH,LAMP1
MKNK1	kinase	0.0195	LAMC1,NELL2,NRXN1,PLTP
CD38	enzyme	0.0209	CHST12,JCHAIN,PKM,SELL,THY1
AHI1	other	0.0227	NTRK2
IGF2	growth factor	0.0227	IGFBP3
FKRP	other	0.0227	LAMC1
HOXA13	transcription regulator	0.0227	EPHA4
MSX2	transcription regulator	0.0227	EPHA4
CDH2	other	0.0227	CDH5
ADAM17	peptidase	0.0227	ICOSLG/LOC102723996
PTK2	kinase	0.0227	VCAM1
LTB	cytokine	0.0227	SELL
LPAR1	g-protein coupled receptor	0.0227	CACNA2D1
TNFRSF11A	transmembrane receptor	0.0227	VCAM1
P2RY2	g-protein coupled receptor	0.0227	VCAM1
ERBB4	kinase	0.0227	CADM1
BDKRB2	g-protein	0.0227	CD44

	coupled receptor		
SREBF2	transcription regulator	0.0227	LRP1
IL4	cytokine	0.0234	CD200,CD33,CD44,JCHAIN,KIT, LGALS3BP,THY1,VCAM1
KLF2	transcription regulator	0.0235	S1PR1,SELL
C3	peptidase	0.0235	C3,SELL
DIO3	enzyme	0.0235	COL6A1,SEMA7A
ARNT	transcription regulator	0.0257	S1PR1,SELL
PSEN1	peptidase	0.0286	AXL,BSG,C3,L1CAM,LRP1,NCAM1,PKM, RELN
GLDN	other	0.0338	NFASC
KCNK2	ion channel	0.0338	VCAM1
TBR1	transcription regulator	0.0338	RELN
NRCAM	other	0.0338	NFASC
NEUROD1	transcription regulator	0.0338	NTRK2
NFIX	transcription regulator	0.0338	SPARCL1
KLF3	transcription regulator	0.0338	SELL
LCP2	other	0.0338	LAMP1
Tnfsf9	other	0.0338	VCAM1
NR2F2	ligand-dependent nuclear receptor	0.0338	NRP1
GFAP	other	0.0338	CD44
HSF2	transcription regulator	0.0338	RELN
CD36	transmembrane receptor	0.0338	LRP1
IKZF1	transcription regulator	0.0351	AXL,LRP1,NCAM1
HDAC4	transcription regulator	0.038	CACNA2D1,CNTN1,NRCAM
IL6	cytokine	0.038	KIT,SERPINA3,VCAM1
IGF1	growth factor	0.0428	IGFBP3,THY1
FCGR3A/FCGR3B	transmembrane receptor	0.0448	SELL
OPRK1	g-protein coupled receptor	0.0448	NRP1
NFIA	transcription regulator	0.0448	EFNB1
DAG1	transmembrane	0.0448	CDH5

	receptor		
NGFR	transmembrane receptor	0.0448	NCAM1
F3	transmembrane receptor	0.0448	VCAM1
SPTBN2	other	0.0448	NCAM1
MSX1	transcription regulator	0.0448	EPHA4
CLEC4M	other	0.0448	C3

Regulated proteins were exported and used for pathway analysis using the Ingenuity Pathway Analysis program
